# Free SARS-CoV-2 Spike Protein S1 Particles May Play a Role in the Pathogenesis of COVID-19 Infection

**DOI:** 10.1134/S0006297921030032

**Published:** 2020-12-30

**Authors:** Andrey V. Letarov, Vladislav V. Babenko, Eugene E. Kulikov

**Affiliations:** 1 Winogradsky Institute of Microbiology, Biotechnology Research Center, Russian Academy of Sciences, 117312 Moscow, Russia; 2 Faculty of Biology, Lomonosov Moscow State University, 119991 Moscow, Russia; 3 Federal Research and Clinical Centre of Physical-Chemical Medicine, Federal Medical Biological Agency, 119435 Moscow, Russia

**Keywords:** SARS-CoV-2, renin-angiotensin system, spike protein, S1 subunit shedding, COVID-19 pathogenesis, D614G mutation

## Abstract

The imbalance of the renin-angiotensin system is currently considered as a potentially important factor of the pathogenesis of COVID-19 disease. It has been shown previously in the murine model, that the expression of angiotensin-converting enzyme 2 (ACE2) on the cell surface is downregulated in response to the infection by SARS-CoV virus or recombinant spike protein (S protein) alone. In the case of natural infection, circulation of the S protein in a soluble form is unlikely. However, in SARS-CoV-2, a large fraction of S protein trimers is pre-processed during virion morphogenesis due to the presence of furin protease cleavage site between the S1 and S2 subunits. Therefore, S protein transition into the fusion conformation may be accompanied by the separation of the S1 subunits carrying the receptor-binding domains from the membrane-bound S2 subunits. The fate of the S1 particles shed due to the spontaneous “firing” of some S protein trimers exposed on the virions and on the surface of infected cells has been never investigated. We hypothesize that the soluble S1 subunits of the SARS-CoV-2 S protein shed from the infected cells and from the virions *in vivo* may bind to the ACE2 and downregulate cell surface expression of this protein. The decrease in the ACE2 activity on the background of constant or increased ACE activity in the lungs may lead to the prevalence of angiotensin II effects over those of angiotensin (1-7), thus promoting thrombosis, inflammation, and pulmonary damage. This hypothesis also suggests the association between less pronounced shedding of the S1 particles reported for the S protein carrying the D614G mutation (vs. the wild type D614 protein), and lack of increased severity of the COVID-19 infection caused by the mutant (D614G) SARS-CoV-2 strain, despite its higher infectivity and higher *in vivo* viral load.

## INTRODUCTION

The COVID-19 pandemic that has already resulted in about two million fatalities worldwide is caused by the SARS-CoV-2 coronavirus, that was most probably transmitted from an animal host to humans late in 2019 [1]. SARS-CoV-2 is closely related to the bat coronavirus RaTG13; however, the existence of the transient host (probably, a pangolin) was suggested [2]. It is believed that SARS-CoV-2 adaptation to the human host occurred mostly *via* acquisition of the novel sequence of the receptor-binding domain (RBD) of the tail spike (S) protein that efficiently recognizes human angiotensin-converting enzyme 2 (ACE2). Although related SARS-CoV virus also recognizes ACE2 as its cellular receptor [1], only 8 out of 14 amino acid (a.a.) residues involved in the RBD interaction with ACE2 are conserved between SARS-CoV and SARS-CoV-2 [2].

The entry of the coronavirus into the host cell requires the cleavage of the S protein at the junction between the S1 and S2 subunits by host proteases [1]. This cleavage can take place after virion attachment (by the cell surface protease TMPRSS2) or in the lysosomal compartment after the virus internalization [1, 3]. The proteolytic processing of the S protein allows the S1 subunit to dissociate, triggering the S2 subunit rearrangement into its extended conformation required for the initiation of the fusion of the viral and lysosomal membranes [4].

In some coronaviruses, however, the processing of the S protein takes place during the virion assembly in the Golgi complex and requires the presence of a recognition site for furin protease at the junction between the S1 and S2 subunits [3]. In SARS-CoV-2, insertion of the 4-a.a. sequence PRRA after a.a. 675 in the S protein has created the furin cleavage site RRAR [2], ensuring efficient processing of the S protein in the virus-producing cell [3, 5]. The pre-processing of the S protein before the virus release makes infection more efficient [3] and potentially may allow some of the viral particles to penetrate into the host cells directly through the plasma membrane without entering the lysosomal compartment [6]. A more efficient SARS-CoV-2 entry into the host cells, together with a higher affinity of its RBD for the receptor (compared to SARS-CoV RBD which also lacks the furin cleavage site) may compensate for a decreased availability of the SARS-CoV-2 RBDs for the ACE2 binding [3]. Indeed, in most trimers of theSARS-CoV-2 S protein, two out of three RBDs remain in the closed conformation, which shields the RBDs from host immunity factors (e.g., antibodies) but also prevents the receptor recognition, while in SARS-CoV, all three RBDs are in the “open” conformation on most of the spikes [7].

As in all viral pneumonias, the pathology of COVID-19, as well of SARS, is determined by the virus-meditated destruction of lung epithelial cells, and, even to a greater extent, by the side effects of the immune system response to the infection [1, 8].

## HYPOTHESIS AND DISCUSSION

However, the fact that both SARS-CoV and SARS-CoV-2 recognize ACE2 as a receptor has given rise to the hypothesis that in addition to the aforementioned mechanisms (which may be further exacerbated by the secondary bacterial infection), the pathology of COVID-19 or SARS may be to a large extent associated with the virus-induced imbalance of the renin-angiotensin system (RAS) [9, 10].

ACE2 is a cell-surface metalloprotease (carboxypeptidase) that converts angiotensin I decapeptide into angiotensin (1-9) nonapeptide, in contrast to ACE1 (or ACE) enzyme, which converts angiotensin I to physiologically active angiotensin II [or angiotensin (1-8)] octapeptide. Angiotensin (1-9) generated by ACE2 can be further processed into the angiotensin (1-7) heptapeptide by the ACE1 enzyme. Angiotensin II, in turn, may be processed into angiotensin (1-7) by ACE2 [9, 10]. Under normal physiological conditions, angiotensin II increases arterial pressure due to vasoconstriction; at the same time, it promotes local inflammation, blood coagulation, thrombosis, and fibrosis, enhances capillary permeability, and causes edema. Angiotensin (1-7) has the opposite effect, i.e., it decreases inflammation, thrombosis, and fibrosis and causes vasodilatation [9, 10]. Therefore, the upregulation of the cell surface expression of ACE1 and/or downregulation of the cell surface expression of ACE2 can lead to more pronounced pulmonary damage.

It has been demonstrated that the SARS-CoV infection in mice decreases the cell surface expression of ACE2 [9-11]. It was suggested that the virus attachment to the ACE2 molecules causes their removal from the cell surface *via* co-endocytosis with the virus, thus diminishing the ACE2 activity. This leads to the imbalance between angiotensin II and angiotensin (1-7) in the lung tissue and promotes thrombosis and pulmonary damage [9, 10].

We believe that direct mechanical removal of the ACE2 molecules by the virion attachment is unlikely to affect significantly the overall activity of this enzyme in the lungs, as it would require simultaneous virus attachment to a large fraction of ACE2-producing cells (such as alveocytes II). The direct damage of the epithelial cells at such a high viral load would be incompatible with the patient survival. However, the reported mortality even in severe COVID-19 cases is moderate [1, 8] and, to our knowledge, no COVID-19 cases with extreme viral load have been reported so far.

Interestingly, the authors of [11] demonstrated that reduction of the ACE2 level in mice could be induced not only by the SARS-CoV infection, but also by the recombinant SARS-CoV S protein. The mice pre-treated (i.p.) with this spike protein did not show significant pathology, however when these animals were experimentally instilled with acid, the spike protein pre-treatment led to increased severity of the lung damage [11]. Therefore, been synthetized in a significant molar excess relative to the viral particles, the S protein may in fact mediate the downregulation of the ACE2 cell surface expression and RAS imbalance. It has been shown that the S proteins of murine coronaviruses and of SARS-CoV are delivered onto the cell surface, presumably, as side products of the virus assembly and release. These molecules may induce some physiological effects, such as micropinocytosis and/or membrane fusion of the neighboring cells [6]; however, the S protein always remains attached to the infected cell membrane. It worth to mention that multiple copies of the S protein are also present on the surface of viral particles released from the infected cells into the medium.

It has been recently demonstrated by direct cryo-electron and transmission electron microscopy [12] that most of S protein trimers on the surface of *in vitro* cultured SARS-CoV-2 particles exist in the post-fusion conformation. In other words, the dissociation of S1 subunits takes place before the receptor binding. Although in this study, dissociation of the S1 subunits could in part be caused by the gradient centrifugation used to purify the virus or by the virus inactivation with 0,05% β-propiolactone [12], the result demonstrates that dissociation of the S1 subunit in the processed SARS-CoV-2 spikes can be triggered by relatively mild conditions or occur spontaneously. The shedding of the S1 subunits from the viable lentivirus particles pseudotyped with the SARS-CoV-2 S protein has also been demonstrated recently [13], confirming the possibility of spontaneous “firing” of the S protein.

We hypothesize that infected cells and virions can shed substantial amount of free soluble S1 subunits (figure).

**Figure.**
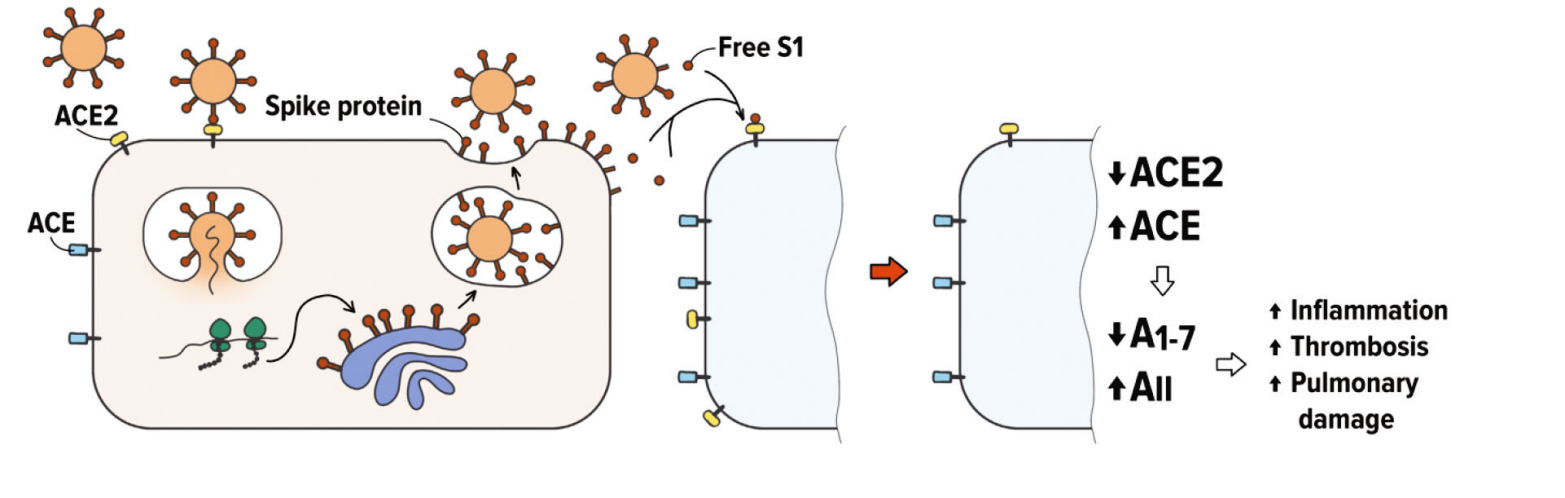
Putative involvement of free S1 subunits of the SARS-CoV-2 S protein in the COVID-19 infection. Spontaneous “firing” of the S protein trimers on the surface of virions and infected cells liberates free RBD-containing S1 particles. The binding of these S1 particles to ACE2 may cause a decrease in the ACE2 cell surface expression and lead to the RAS imbalance.

S1 molecules carry intact RBD domains, and their binding to ACE2 may induce ACE2 downregulation and deleterious downstream effects, as it was suggested earlier in [9, 14].

If our hypothesis is true, the release of free S1 particles from the infected cells and virions should reduce the virus infectivity towards the neighboring cells by decreasing the number of ACE2 receptor molecules on their surface. Therefore, two stages of the SARS-CoV-2 infection in the lungs can be predicted:

1) The virus infects certain loci, in which production of free S1 protein causes ACE2 downregulation of in the non-infected cells in the proximity of the virus-infected cells. Simultaneously, ACE2 expression on more distant cells (those that have not yet encounter S1 particles) can be increased due to the effect of interferon [15] produced by the infected cells. The local imbalance of the angiotensin II/angiotensin (1-7) levels also may elicit compensatory ACE2 increase in the cells not affected by the virus (although such compensatory circuit has not yet been described, it would be logical to suggest it may exist). Therefore, the spread of the virus at larger distances (e.g., to other alveolae) will be facilitated. At the same time, the virus-induced tissue damage would be limited.

2) When larger volume of the tissue is infected, free S1 is produced in greater quantities, and the RAS imbalance is induced at the organ or organism levels, resulting in such deleterious effects as increased inflammation, thrombosis, and pulmonary damage (as suggested previously in [9, 14]). Simultaneously, the virus production will be decreased due to the downregulation of ACE2 expression in the infected areas of the lungs.

This model corresponds well to the clinical observations in the COVID-19 cases [16, 17]: The pathological process often develops rapidly and involves large areas of the lungs with frequent bilateral pneumonia (stage I of our model). At the same time, the patients frequently feel relatively well despite the fact that a significant part of their lungs is affected according to radiological examination data. This may be followed by an abrupt deterioration of the patient’s condition in few hours or days (late stage I and stage II).

The proposed model predicts that the previously suggested clinical interventions aiming to maintain the RAS balance [18] would have synergistic effect with protease inhibitors, especially, with furin inhibitors. Although *in vitro* data indicate that the lack of furin pre-processing of the SARS-CoV-2 S protein can be compensated by the post-attachment cleavage by other proteases [3], our model suggests that furin inhibition will not only reduce the virus infectivity but will also decrease the shedding of free S1 particles from both the virions and the infected cells, thus alleviating the tissue damage induced by the RAS imbalance.

Recently, SARS-CoV-2 isolates with the D614G mutation in the S protein were described [13, 19-22]. Over a short period of time, those mutant variants have gained dominance in many areas where they were found. It was demonstrated that the D614G mutation decreases the stability of the S protein trimer and facilitates RBD transition into its open conformation [23]. This mutation increases the overall virus infectivity and fitness [19-22, 24], increases affinity of furin binding to the S protein [25], and promotes viral replication [26]. However, there are no indications that the D614G mutation is associated with more severe COVID-19 infection symptoms. Surprisingly, despite the fact that this mutation destabilizes the trimer, it was shown that the S1 shedding from the lentiviruses pseudotyped with the D614G mutant SARS-CoV-2 S protein [13] was significantly reduced compared to the wild-type D614 protein. Our hypothesis suggests that the decreased S1 shedding may be one of the factors limiting the morbidity and mortality of the D614G SARS-CoV-2 infection despite a higher infectivity of the virus and higher viral loads observed for the mutant virus [20].

The experiments to test our hypothesis on theS1-mediated pathogenesis of COVID-19 infection could be relatively simple and straightforward, and may include quantitative measurements of the concentration of S1 particles in the SARS-CoV-2-infected cell cultures, isolation of S1 particles by ultrafiltration of the cell culture supernatant, experimental testing of the binding of these particles to the ACE2 receptor, and evaluation of the effects of S1 particles on the ACE2 expression and RAS parameters in cell cultures and animal models.

It should also be mentioned that free S1 molecules may represent a target for the COVID-19 therapy or prevention. The separation of the S1 subunit from the S2 subunit exposes potential epitopes that are free of glycans, which normally shield the external surface of the complete S protein [27]. So, immunization with the recombinant proteins that would elicit the antibody response against these conserved and unprotected epitopes may lead to the sequestration of free S1 molecules into immune complexes and their subsequent elimination, which would reduce the probability of severe COVID-19 pneumonia.

## Abbreviations

ACE: angiotensin-converting enzyme
RAS: renin-angiotensin system
RBD: receptor-binding domain
S protein: spike protein
SARS-CoV: severe acute respiratory syndrome coronavirus
